# Aneurysmectomy with Partial Nephrectomy on a Living Donor Renal Allograft: A Case Report

**DOI:** 10.1155/2013/791413

**Published:** 2013-02-21

**Authors:** Siegfredo Paloyo, Junichiro Sageshima, Linda Chen, George W. Burke, Gaetano Ciancio

**Affiliations:** ^1^Division of Transplantation, Department of Surgery, Leonard M. Miller School of Medicine, University of Miami, Miami, FL 33136, USA; ^2^Miami Transplant Institute, 1801 NW 9th Ave., Highland Professional Building, Miami, FL33136, USA

## Abstract

Vascular anomalies among living kidney donors are seldom encountered and their presence offers a complex opportunity for every transplant surgeon. Furthermore, there has been an increasing trend with the use of marginal or kidneys with pathology to address the shortage of organs. We report a rare case of a kidney allograft with a saccular aneurysm and renal cortical cysts for which an excision with primary repair and partial nephrectomy were done, respectively. The recipient was a 45-year-old female with lupus nephritis and significant comorbidities who had excellent recovery and outcome. With good surgical techniques, these types of grafts continue to provide acceptable outcome but safety of the donor should be of utmost importance.

## 1. Introduction

Renal artery aneurysms are rare occurrences whose incidence ranges from 0.1 to 1%, of which 80% is saccular in form [1]. There are only a few reports of this pathology in the transplant setting. Its management mainly involves excision with primary closure or use of a prosthetic or native vessel patch. Excellent short- and long-term graft outcomes have been documented with these surgical techniques [1–8]. We report a unique case of a saccular aneurysm found incidentally on a living kidney donor associated with renal cortical cysts, which, when approached surgically, could potentially compromise renal graft function after transplant.

## 2. Case Report

**Figure 1:**
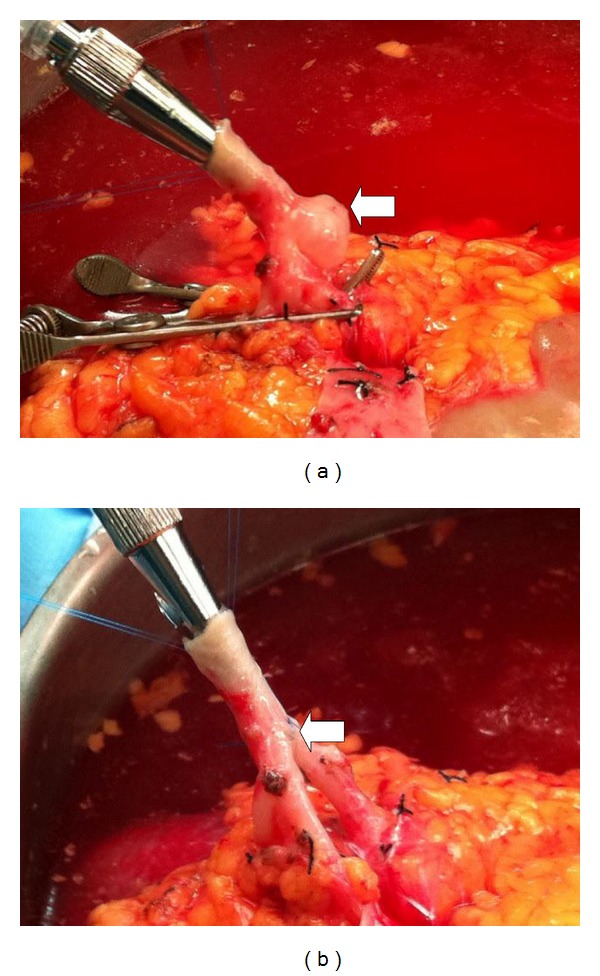
Saccular aneurysm of the donor renal artery before (a) and after (b) vascular reconstruction (*arrow*).

A 45-year-old Caucasian female with end-stage renal disease secondary to lupus nephritis underwent a living donor kidney transplant with her husband (1-DR match) as her donor. Significant past medical history included a colon resection for perforation secondary to a peritoneal dialysis catheter complicated by an intra-abdominal abscess, preeclampsia, debridement, and skin grafting on the lower extremities for extensive fungal infection, hypertension, glaucoma, and a previous highly sensitized state due to multiple pregnancies and blood transfusions for which she underwent desensitization. A preoperative CT scan of the right kidney allograft showed an upper pole cyst measuring 4 × 3.5 × 2 cm and an inferior pole cyst measuring 2 × 1.5 × 1 cm. The donor subsequently underwent an uncomplicated right donor laparoscopic nephrectomy. While preparing the allograft on the back table, it was noted that there was a 1 × 0.5 × 0.2 cm saccular aneurysm on the bifurcation of the renal artery. A simple excision and primary closure was performed on this after meticulous dissection (Figure 1).

**Figure 2:**
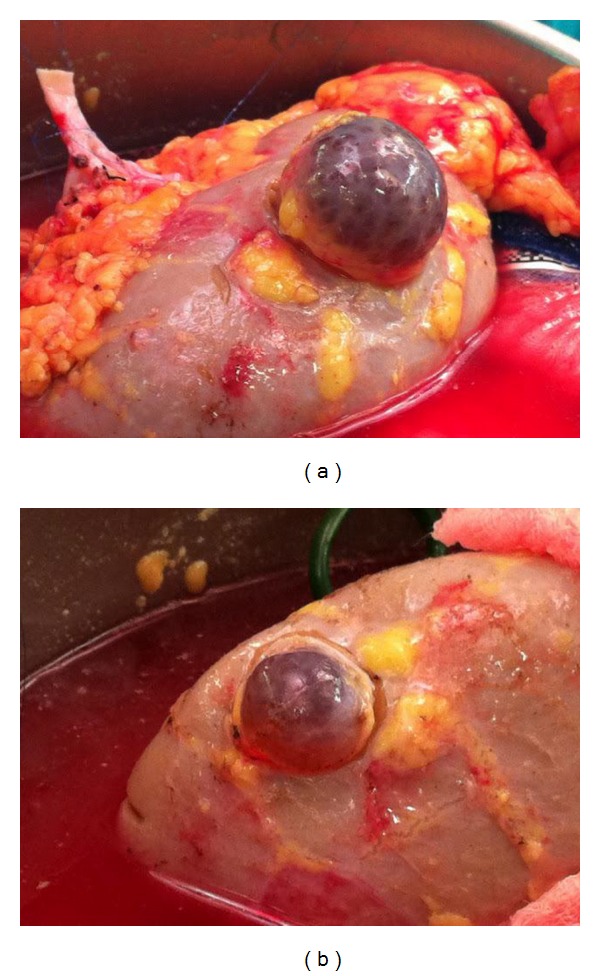
Renal cortical cysts located in the upper pole (a) and lower pole (b) of allograft.

We then proceeded with a partial nephrectomy for the 2 renal cortical cysts (Figure 2). After all the preparation, the renal allograft was implanted in the right iliac fossa, anastomosed end-to-side to the external iliac vessels, and an extravesical ureteroneocystostomy for bladder anastomosis was performed. Postoperative course was uncomplicated and the patient had excellent diuresis. She was discharged after 9 days with a creatinine of 2 mg/dL from a baseline of 6.3 mg/dL. The surveillance ultrasound showed good flow for both the renal artery and vein 2 months after surgery with a creatinine of 1.4 mg/dL.

## 3. Discussion

The use of marginal kidneys and grafts with anatomical abnormalities has been increasingly reported due to the lack of organs [3]. Accumulated experience and innovative techniques acquired by transplant surgeons have provided excellent outcomes with minimal complications. As exemplified by this case, the rarity of finding a vascular abnormality together with a common renal pathology on a living donor can be very challenging indeed.

**Figure 3:**
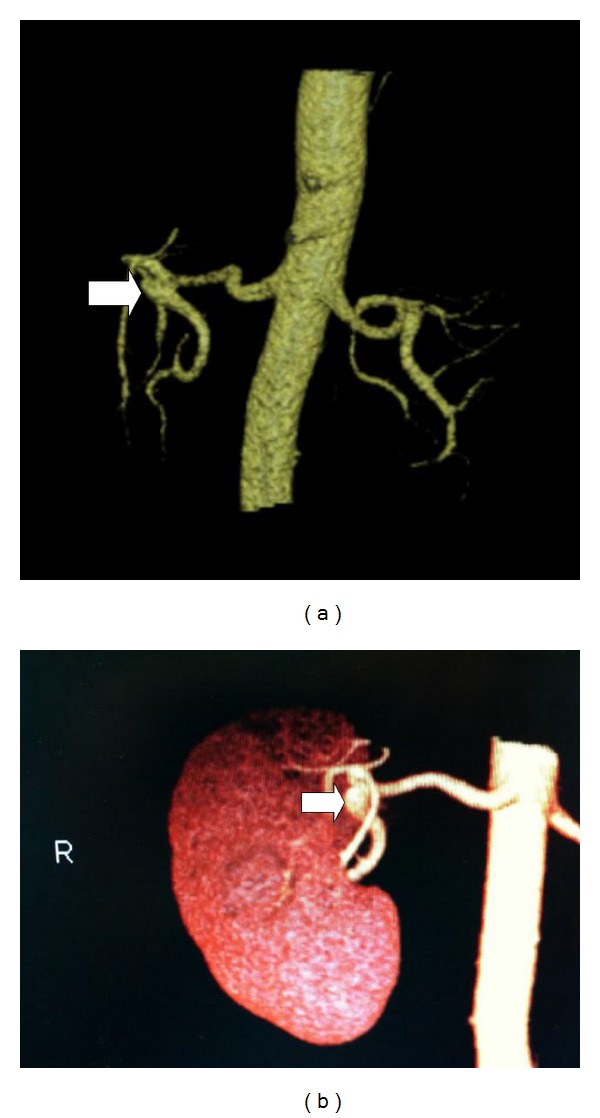
CT angiogram with 3D reconstruction shows a saccular aneurysm arising from the proximal right lower pole renal artery at the bifurcation of the main renal artery (*arrow*).

A CT angiogram has become the standard method of evaluating potential living kidney donors with reported sensitivity and specificity for arterial anatomy of 91 and 93%, respectively [9]. Infrequently, vascular anomalies are missed which are almost always visible when viewed retrospectively. Upon review of the CT arteriogram with 3D reconstruction, the aneurysm was clearly identified which was initially interpreted as a tortuosity in the renal artery (Figure 3). 

Olakkengil and Mohan Rao reported a series of 6 donors, both living and deceased, with renal artery aneurysms mostly performing an excision and primary closure with favorable outcomes, although 1 recipient had graft loss after a year which was unrelated to the vascular reconstruction [1]. They also noted the importance of communication between the retrieval and transplant team especially in deceased donors because this pathology can often be missed due to the collapsed arterial system and lack of preoperative imaging studies. Another series by Toda et al. reported on 7 cases also describing the aforementioned technique with angioplasty and clipping of the aneurysm as additional surgical approaches [2]. The longer total ischemic time (average of 2 hours) required for vascular repair did not affect postoperative graft function.

To our knowledge, this is the first such case to be reported in the literature. Based on its size, the aneurysm was amenable for excision and primary repair *ex vivo* after full exposure and dissection. Considering the added ischemia time with the vascular repair and decrease in nephron mass after partial nephrectomy, the renal allograft was deemed adequate to support the metabolic needs of the recipient. Evidently, the patient had immediate diuresis with a reasonable creatinine upon discharge. It is important to keep in mind that restoring lesions close to the hilum sometimes requires extensive perihilar dissections which can lead to lymphatic leaks or bleeding after release of clamps.

In conclusion, surgically correctable abnormalities on renal allografts, either as a single pathology or, such as this case, in combination, are able to provide excellent outcomes once they are repaired. However, while the use of these grafts are safe, careful donor screening cannot be over emphasized to ensure that the remaining kidney is normal.
